# The influence of artificial saliva on the cleaning force of interdental rubber picks: an in-vitro comparison

**DOI:** 10.1186/s12903-022-02479-6

**Published:** 2022-11-01

**Authors:** Christian Graetz, Ann-Kristin Härdter, Susanne Schorr, Miriam Cyris, Antje Geiken, Thomas Rinder, Christof E. Dörfer, Sonja Sälzer

**Affiliations:** 1 Clinic of Conservative Dentistry and Periodontology, University of Kiel, Arnold-Heller-Str. 3, Haus B, 24105 Kiel, Germany; 2 Institute of Mechatronics, Computer Science and Electrical Engineering, Kiel University of Applied Sciences, Kiel, Germany

**Keywords:** Oral hygiene, Interdental brushes, Mechanical plaque control, Interdental cleaning force

## Abstract

**Background:**

The familiar aids for interdental cleaning such as dental floss or interdental brushes (IDB) are often associated with difficult handling or an increased potential for trauma. Interdental picks (IRP), which have no metal core and silicone flaps instead of nylon brushes, offer the alternative. However, in-vitro studies found a lower cleaning effectiveness combined with higher forces for cleaning compared with conventional IDBs. The aim of this in-vitro study was to measure the experimental cleaning forces (ECF) using IRP with versus without an artificial saliva (AS; GUM Hydral, Sunstar Suisse SA, Etoy, Switzerland).

**Methods:**

The test set-up was developed to investigate the cleaning of 3D-printed interdental area (IDR) mimicking human teeth (Form 2, Formlabs Sommerville, MA, USA) under standardized conditions. Three different morphologies (isosceles triangle, convex, concave) and three different sizes (1.0 mm,1.1 mm,1.3 mm) were used. Two different IRPs (GUM Soft-picks Advanced: SPA versus GUM Soft-picks Advanced Plus: SPA+, Sunstar Suisse SA, Etoy, Switzerland) in three sizes (small, regular, large), were used with versus without AS. ECF during ten cleaning cycles were recorded by a load cell [N].

**Results:**

Using AS leaded to significant lower values for ECF than without (1.04 ± 0.66 N versus 1.97 ± 1.01 N, p < 0.001). In general, a lower ECF was recorded for convex IDR compared to isosceles triangle and concave morphologies (p < 0.001) as well as for gap sizes of 1.3 mm compared to the smaller sizes (p < 0.001). For SPA+ we found significantly higher force values than for SPA (1.67 ± 0.93 N versus 1.31 ± 0.97 N, p < 0.001) independent of the use of AS.

**Conclusion:**

Within the study´s in-vitro limitations, we found AS reduced ECF of IRPs by half and allowed using larger diameters interdentally, which could be associated with (1) a higher cleaning effectiveness and (2) a higher acceptance e.g. of patients with dry mouth. This has to be confirmed by further clinical investigations.

## Background

Mechanical cleaning applies to the most important and common means to amplify the tooth surface of inherent microbial biofilm. By routine and systematic tooth brushing plaque can be removed and common oral diseases such as caries, gingivitis or periodontitis can be prevented. However, cleaning efficacy with a normal toothbrush reaches in mean 60% [17] and one of the reasons is that the bristles of toothbrushes merely reach the interdental area (IDR) which is the most predestined location of caries and gingivitis [11].

Therefore, numerous cleaning aids such as dental floss or interdental brushes (IDB) were developed as support for interdental cleaning. Regarding the cleaning efficacy and the adaption to the interdental space, IDB have the best outcome whereas dental floss only results in a lower reduction of plaque [5, 15, 16, 23, 25]. However, many patients avoid IDB for their difficult handling, soft-tissue trauma [9] and the risk of breakage or deformation of the metallic core and prefer to use dental floss. This, certainly has not as much effect on plaque removal as IDBs, because of the lack of adaptation to the interdental space morphology and the more cumbersome handling for patients [13].

Overcoming the limitations of IDBs, a new tool was developed as a kind of advancement - the interdental rubber picks (IRP). Unlike IDBs, these do not require a metal core and are said to be easier to use than dental floss, without a corresponding risk of breakage or trauma. Clinical trials demonstrate high patient acceptance [1, 15] and an actual systematic analysis found comparable results for gingivitis treatment with IRPs or IDBs [19]. However, in-vitro studies showed significantly lower cleaning performance with increased force [8, 21]. As a result, our science group discussed efforts to improve the cleaning effectiveness by further developing the shape and material of the IRP. Although this resulted in better cleaning efficiency, the forces involved in the cleaning process increased even more.

Several in-vitro studies compared different interdental cleaning aids including IRPs, nevertheless, oftentimes the experimental method neglected the variety of human tooth morphologies in IDR [18, 21, 22] and to our best knowledge, all of them tested under dry conditions, which only represents a small spectrum of patients with dry mouth and not the majority. So, we reconsidered our in-vitro model and concluded that the lack of saliva results in higher force values. Saliva has a prominent role in the oral cavity: in addition to the antibacterial effect, buffering and clearance, remineralization of the teeth and the participation in tasting and digestion, particularly the function as a lubricant for the sliding of the soft tissues against each other and against the teeth plays a key function [10]. The viscosity and the gliding property of saliva is generated by certain mucins and glycoproteins secreted mainly by the minor salivary glands. Artificial saliva can replace and mimic some of these important properties. In addition, certain contained anti-caries bioactive components contribute to protection against caries or gingivitis by inducing remineralization [12].

Therefore, the aim of the current in-vitro study was to measure the experimental cleaning forces (ECF in N) with versus without artificial saliva (AS) for different types of IRP. The primary hypothesis is that the ECF values measured on the dry model will be significant higher compared with that of using AS.

## Methods

### Experimental setup

As the model basis, the experimental device developed in previous publications [7, 8] was modified in such a way, that a situation adapted to the oral cavity is possible under reproducible conditions. Therefore, the current in-vitro model simulates even more realistically a patient situation of oral hygiene at home, i.e. for patients with dry mouth. As explained in detail in our previous publications [7, 8] we used 3D printed replicas of human teeth to create different intraoral morphologies by a computer software (Autodesk Fusion 360, Autodesk Direct Limited, Hampshire, United Kingdom) which were printed by a 3D printer (Form 2, Formlabs Sommerville, MA, USA) in a stereolithography way by using liquid photopolymer resin (White Resin V04 (RS-F2-GPWH-04), Formlabs, Sommerville, MA, USA).

We used artificial IDR of three gap sizes of 1 mm (small), 1.1 mm (medium) and 1.3 mm (large) and three different morphologies (isosceles triangle, concave and convex) giving us the chance to make our tests with nine different examples of IDR.

The corresponding replicas were fastened in a socked that was connected to an embedded load cell (KD34s, ME-Meßsysteme GmbH Hennigsdorf, Germany; measuring range: ± 500 mN with precision class of 0.1%) enabling us to track all emerging forces during the cleaning process and to document them automatically in a table (Microsoft Excel 2016, Microsoft Corporation, Redmond, WA, USA). Due to possible background noises the values ≤ 0.1 N were not included.

**Fig. 1:**
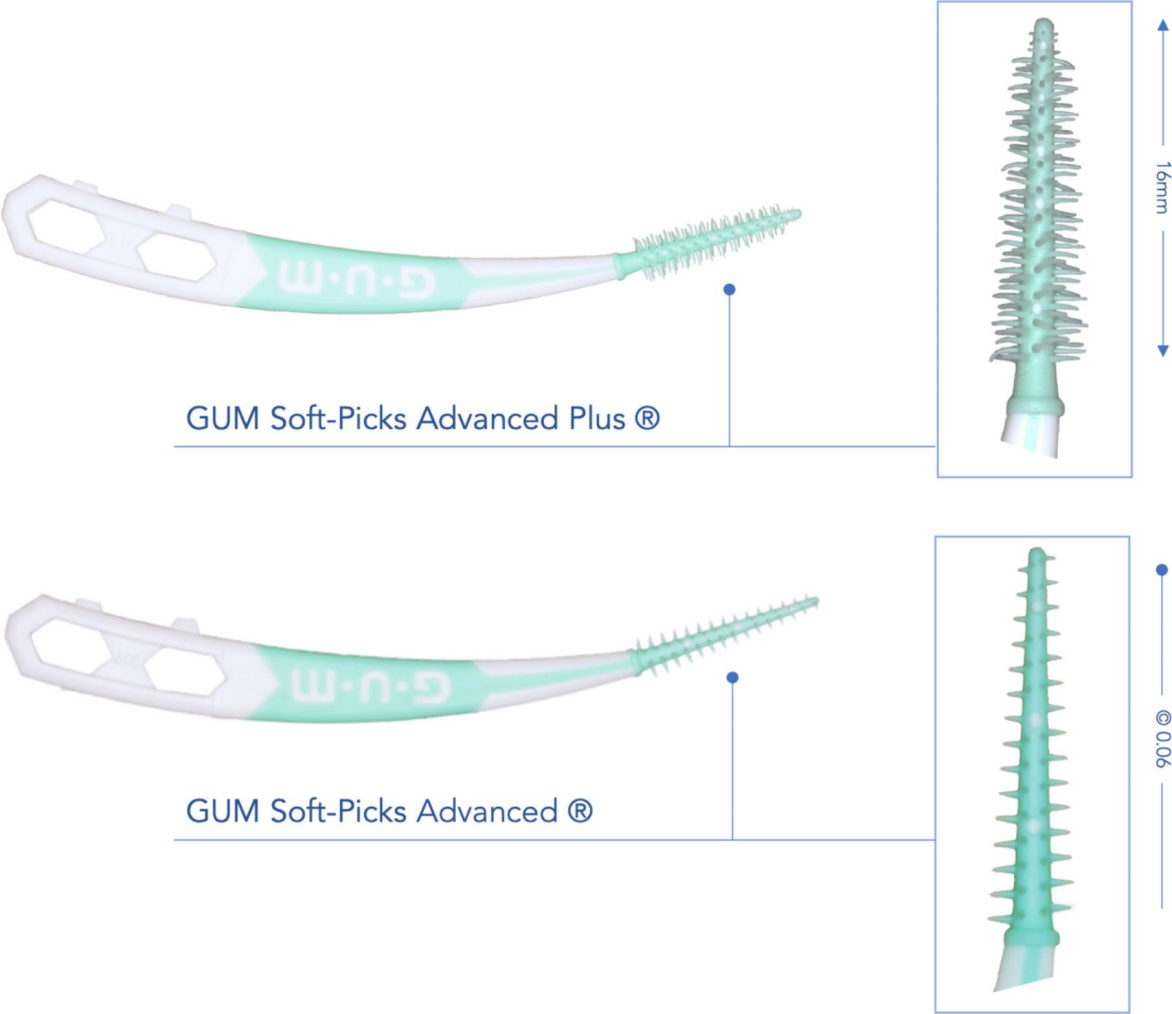
Illustration of the tested cleaning devices in regular size. The working part has 16 mm length with a taper of 0.06 of the core. (Test products: GUM Soft-Picks Advanced (SPA) and GUM Soft-Picks Advanced Plus (SPA+))

As cleaning device (Fig. 1), we used two different types of interdental rubber picks (GUM SOFT-PICKS® Advanced (SPA) and Advanced Plus (SPA+), Sunstar Suisse SA, Etoy, Switzerland). They have a taper of 0.05 and we used them respectively in three different sizes (small, regular, large). These IRPs were classified as “fitted”, “too big” and “too small” based on the IDR as the calibrated examiner A.-K.H. weighed and adjusted the manual force required to insert the IRP into the IDR according to previously published procedure [7, 8] and on the basis of criteria such as the maximum cleaning force < 5 N and the standardized insertion length of 10 mm inner the artificial IDR.

With respect to our aim to test the devices under more physiological conditions like in the oral cavity we used a symptom-relieving moisturizing product (GUM HYDRAL® Moisturizing Spray, Sunstar Suisse SA, Etoy, Switzerland) which contains the properties of artificial saliva that are important for the experiment, such as viscosity and moistening of the interdental space. Therefore, at the beginning of the ten cleaning cycles the IRP were dipped in a vessel of artificial saliva with the whole working length of 16 mm for one second, so the cleaning area of the IRP was fully moistened. A standardized moisture thickness was ensured by a standardized procedure and appropriate time protocol.

For measurement the IRP were fixed in a technical device turning rotation in horizontal movement. For ten cleaning cycles it moved the IRP forwards and backwards into the artificial IDR with a controlled speed while the load cell was documenting all upcoming forces. ECF (in Newton), the force for cleaning the IDR by pushing and pulling the IRP into the gap was calculated as the average value of force (mean ± SD) of ten cleaning cycles. Due to the avoidance of in-vivo long-term damage to the tooth caused by the constant application of excessive forces and the fragility of our load cell, we declared 5 N as a maximum force during cleaning process. As the movement and the measurement stopped immediately at higher force values ≥ 5 N (computer controlled) we failed to show results for these tests.

It should be noted, by using AS in our model basis, it isn’t possible to measure the cleaning efficiency with powder or lack due to a blurring or sticking of the simulated biofilm.

### Statistical analysis

For the sample size we adopted the values determined by a power calculation in our previously published in-vitro study [8]. In keeping with this calculation, we established n = 25 samples per group. The statistical analysis was done with SPSS Statistics (SPSS Statistics 24, IBM, Chicago, IL, USA). We tested all data for normal distribution with the Kolmogorov–Smirnov/Lilliefors test and found that there was no normal distribution for all data (p < 0.001).

We compared the mean values of ECF of all different test products, product sizes as well as the interdental gap sizes and morphologies. Subsequently, statistical significance was inquired using the non-parametric Mann-Whitney-U-Test and Kruskal-Wallis-Test. A linear regression was used to examine the relationships between the predictors (type and size of interdental area, type and size of the tested product) and the ECF (dependent variable). The regression coefficients, standard errors (SE), p-values, and 95% confidence intervals (CI) were used as effect estimates. All tests were two-sided and the statistical significance was assumed when p ≤ 0.05 and adjusted with the Bonferroni correction (p = 0.05 / 3 = 0.0167).

## Results

Table 1 shows an overview of the measured values with and without AS. In some of all tests (n = 2700), especially with large product sizes, maximum cleaning forces of more than five Newton occurred and as mentioned before, these values could not be included in the study (n = 325).

**Table 1:** Subgroup results (mean ± SD) of experimental cleaning forces (ECF in N) of all test products.

		ECF in N								
		IDR 1.0 mm			IDR 1.1 mm			IDR 1.3 mm		
		Isosceles triangle	Concave	Convex	Isosceles triangle	Concave	Convex	Isosceles triangle	Concave	Convex
**SPA Small**	Without AS	2.38 ± 0.26**	2.27 ± 0.31**	1.27 ± 0.18	1.26 ± 0.18	1.72 ± 0.28***	0.89 ± 0.16	0.76 ± 0.09	1.46 ± 0.31*^,^ ***	0.39 ± 0.07
	With AS	0.77 ± 0.03	0.90 ± 0.05	0.34 ± 0.03***	0.44 ± 0.05	0.38 ± 0.02**	0.37 ± 0.02**^,^ ***	0.31 ± 0.07	0.48 ± 0.03	0.17 ± 0.01
**SPA Regular**	Without AS	2.92 ± 0.26**	2.49 ± 0.44**	1.34 ± 0.21	1.86 ± 0.13**	1.89 ± 0.18**	0.98 ± 0.14	0.95 ± 0.09	1.31 ± 0.19	0.56 ± 0.25
	With AS	1.24 ± 0.08**	1.21 ± 0.06**	0.41 ± 0.02***	0.69 ± 0.05**	0.71 ± 0.04**^,^ ***	0.43 ± 0.01***	0.44 ± 0.04	0.68 ± 0.02***	0.21 ± 0.01
**SPA Large**	Without AS	n.a.	n.a.	3.63 ± 0.33	n.a.	n.a.	2.91 ± 0.31	2.72 ± 0.34**	n.a.	1.91 ± 0.18
	With AS	n.a.	n.a.	1.23 ± 0.05	2.47 ± 0.27	1.64 ± 0.05	1.01 ± 0.04	1.12 ± 0.07	2.04 ± 0.06	0.76 ± 0.02
**SPA+ Small**	Without AS	3.52 ± 0.41**	2.97 ± 0.45**^,^ ***	1.62 ± 0.17	2.22 ± 0.17**	2.45 ± 0.29**^,^ ***	1.09 ± 0.15	1.05 ± 0.12	1.48 ± 0.15*	0.64 ± 0.08
	With AS	1.48 ± 0.07	2.06 ± 0.06	0.72 ± 0.03	1.11 ± 0.07**	1.12 ± 0.03**	0.57 ± 0.01	0.53 ± 0.04	0.68 ± 0.02	0.44 ± 0.01
**SPA+ Regular**	Without AS	n.a.	n.a.	1.65 ± 0.26	2.31 ± 0.31**	2.51 ± 0.31**	1.22 ± 0.15	1.07 ± 0.13	1.64 ± 0.13	0.86 ± 0.09
	With AS	1.82 ± 0.04	2.58 ± 0.11	0.90 ± 0.03	1.32 ± 0.04**	1.31 ± 0.06**	0.82 ± 0.03	0.63 ± 0.04	0.88 ± 0.03	0.56 ± 0.02
**SPA+ Large**	Without AS	n.a.	n.a.	3.42 ± 0.26	n.a.	n.a.	2.73 ± 0.31	2.00 ± 0.32	3.40 ± 0.63	1.72 ± 0.27
	With AS	n.a.	n.a.	1.39 ± 0.11	2.78 ± 0.14	2.13 ± 0.13	1.23 ± 0.07	1.03 ± 0.05	1.42 ± 0.08	0.91 ± 0.04

Force during ten cleaning cycles (mean ± SD) for cleaning different types (isosceles triangle, convex, concave) and sizes (1.0 mm, 1.1 mm, 1.3 mm) of the interdental area separated for the tested interdental rubber picks (IRP). We assumed p < 0.05 to be statistically significant (Mann-Whitney-U-Test, Kruskal-Wallis-Test, two-sided with Bonferroni adjustment (p ≤ 0.0167)). Differences between testing with artificial saliva (AS) versus without always significant (p ≤ 0.001)

* no significant difference between SPA versus SPA+

** no significant difference inner group of test product between different types (isosceles triangle, convex, concave)

*** no significant difference inner group of test product between different sizes (1.0 mm, 1.1 mm, 1.3 mm)

Overall, the highest total ECF value was measured without AS in a convex IDR of 1 mm with SPA size large (3.63 ± 0.33 N), whereas the lowest value was also measured in a convex IDR but of 1.3 mm size with SPA size small and with AS (0.17 ± 0.01 N). Without subgrouping for devices or interdental size, the concave interdental space was cleaned with the highest forces, followed by the isosceles triangular and convex (1.77 ± 1.01 N vs. 1.58 ± 0.94 N vs. 1.21 ± 0.88 N; p < 0.001 with Bonferroni-correction). Likewise, the result was influenced by the size of the test products. In this category all sizes of test products had a statistically significant difference in cleaning force and lowest ECF was measured for small IRPs, regular coming in second and large size in third position (1.18 ± 0.82 N vs. 1.28 ± 0.76 N vs. 2.16 ± 1.04 N; p < 0.001 with Bonferroni-correction).

### Comparison of results with vs. without AS

**Fig. 2:**
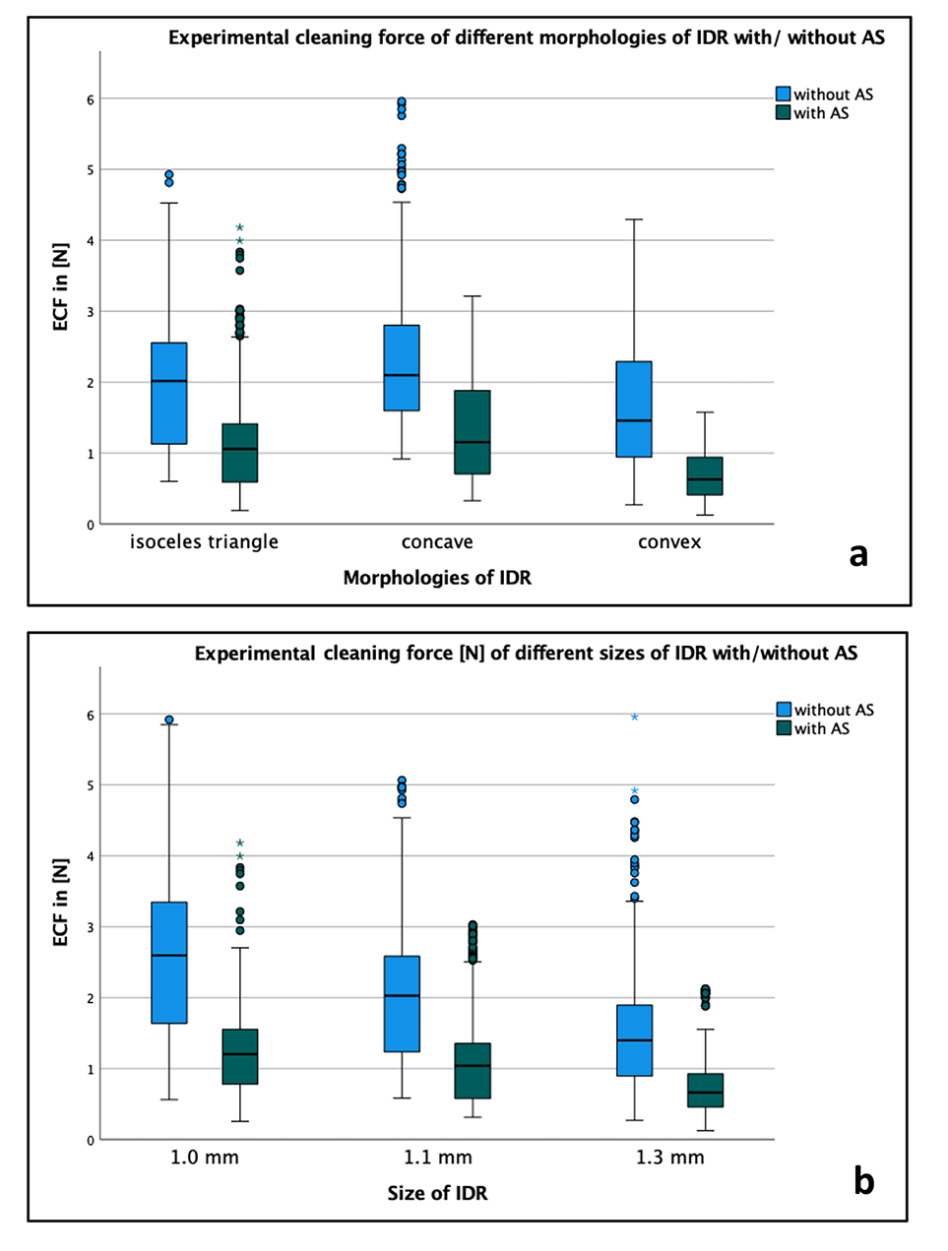
Illustration of the experimental cleaning force (ECF in N) for (**a**) different morphologies of interdental area (IDR: isosceles triangle, concave, convex) and (**b**) for different sizes of the interdental space (IDR: 1.0 mm, 1.1 mm, 1.3 mm) separated for usage of artificial saliva (with/without AS).

In all cases, IRPs had significantly lower cleaning forces when moisture with AS (1.04 ± 0.66 N) than without (1.97 ± 1.01 N; p < 0.001). The largest alteration was discovered brushing the convex IDR with a gap size of 1 mm with SPA in Large (1.23 ± 0.05 N vs. 3.63 ± 0.33 N, p < 0.001) while the smallest difference was in a convex IDR of 1.3 mm with SPA in the small size (0.64 ± 0.08 N vs. 0.44 ± 0.01 N, p < 0.001). With respect to the different morphologies of IDR, the largest difference using AS occurred for the concave IDR (2.37 ± 1.02 N vs. 1.28 ± 0.66 N), followed by the convex and the isosceles triangle IDR (p < 0.001). While without AS statistical differences were found for all morphologies in all sizes (p < 0.001), the use of AS ensured that no significant change in ECF was found between gap sizes 1 and 1.1 mm in the isosceles triangle and convex IDR (p = 0.052 and p = 0.685). With respect to the other categories, we found the greatest change for IDR sizes of 1 mm (2.59 ± 1.00 N vs. 1.28 ± 0.71 N, p < 0.001) and for large product sizes (2.81 ± 0.98 N vs. 1.56 ± 0.68 N, p < 0.001). Using AS, we found a statistical difference between all product sizes (p < 0.001). Figure 2 illustrated the ECF for different morphologies of IDR (Fig. 2a) or different size of IDR (Fig. 2b) separated for usage of artificial saliva (with/without AS).

**Fig. 3:**
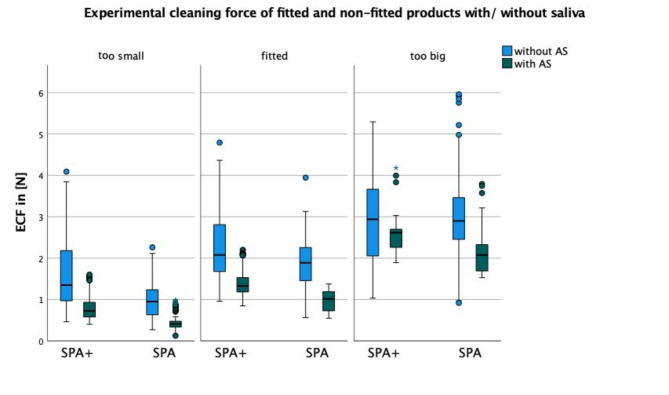
Illustration of the experimental cleaning force (ECF in N) for different sizes of the test products GUM Soft-Picks Advanced Plus (SPA+) and GUM Soft-Picks Advanced (SPA) separated for fitting in the interdental area and usage of artificial saliva (with/without AS).

Dividing the product sizes into fitting IRP for the corresponding IDR and declaring the others as “too big” and “too small”, there is a significant difference in ECF (p < 0.001) with a mean force level for fitted (Table 1; Fig. 3).

### Comparison of different IRP types

When comparing the different IRP types, the ECFs were significantly higher for SPA+ than for SPA (2.11 ± 0.97 N vs. 1.82 ± 1.03 N; p < 0.001). Between product sizes, there was a significant difference of SPA+ and SPA for small and regular size, but not for large size products of both IRPs (p < 0.001 vs. p = 0.290).

## Discussion

The inclusion of interdental cleaning in home oral hygiene is important to prevent carious lesions and periodontal diseases [3, 6]. This results in the challenge of how to motivate patients for daily use of cleaning aids that are as simple as possible. One of the most important factors is the ease of use and pain-free application, which leads to higher acceptance [1, 20]. This is particularly the case for IRP. By optimizing our experimental model, we were able to adapt the test situation much better to the real conditions in the oral cavity. Thus, our primary hypothesis of the current in-vitro study, to show that under the use of AS the measured ECF for IRP was significantly lower and approached ECF values of IDB [18], was confirmed. Mucins and glycoproteins contained in saliva ensure that the soft and hard tissues slide against each other in the oral cavity and are part of the acquired enamel surface [4]. This might be what enables the IRP to slide through the interdental space much more easily and hence reduces ECF.

In contrast, it should be assumed that patients suffering from dry mouth require a much better adaptation considering the higher ECF of IRP in the absence of saliva. Particularly such patients suffer from the loss of protective properties of saliva and are confronted with an increased susceptibility to caries [2, 24], which necessitates the regular use of an effective interdental cleaning aid for domestic oral hygiene. Hence, in patients with xerostomia a possible risk of hard and soft tissue trauma due to the metal core of IDB has to be weighted against its clinical benefits. Further clinical studies will be necessary to evaluate the benefits of each interdental cleaning aid on this special group of patients.

It is important to avoid excessive forces on the adjacent teeth during cleaning, which makes correct fitting of the right IRP size indispensable. The classification of the respective devices into “fitted”, “too small” and “too large” showed a significant difference in ECF (Table 1). While a significant difference was confirmed in all three classifications, the largest was found in measurement of ECF with and without AS in the IRP rated as “fitted”. Thereby it is possible to maximize the contact area of the silicone lamellae on the tooth surface in order to remove as much plaque as possible. Thus, in contrast to the trials without saliva, larger diameters could be classified as suitable in some cases, since the lubricating effect of the AS significantly facilitated sliding through the interdental space what could lead to better results in cleaning efficacy and therefore in better chances to prevent oral diseases. With respect to this result, we were able to confirm a hypothesis of a preprint in-vitro study [14] that choosing a larger ISO size of interdental cleaning aid than the existing embrasure leads to better results, but we can only recommend it for patients with a normal salivary flow. For patients suffering from dry mouth and denture caused stomatitis it would be recommendable to use a dental gel of antibacterial fluids or fluoride together with the IRP so on one hand the occurring ECF would be on an acceptable level and on the other hand the contained anti-caries bioactive components of the gel helps to prevent further demineralization or plaque-induced gingivitis [11].

**Fig. 4:**
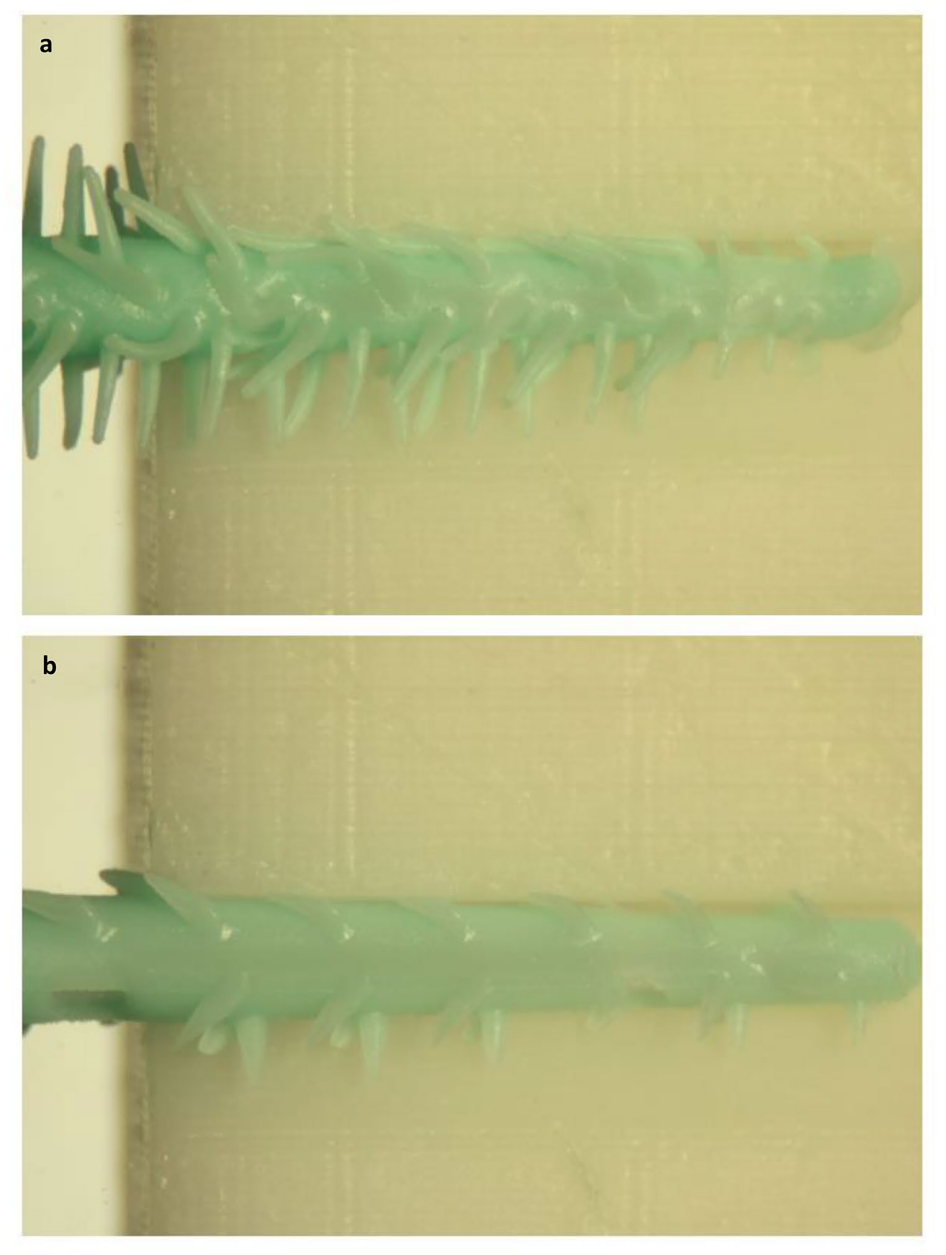
Exemplary illustration of the insertion of the test products (a) SPA+ (GUM Soft-Picks Advanced Plus) and (b) SPA (GUM Soft-Picks Advanced), both in regular size, into an isosceles triangle IDR of 1.3 mm (magnification 2.7x).

The second aim of the study was to compare two different types of IRP. As mentioned, the SPA+ showed overall significantly higher ECF values. This could be explained by the lower ECF so even bigger sizes of SPA could be used. In addition, we measured the highest ECF for fitted SPA+ in the concave interspace, while for fitted SPA the isosceles triangle IDR the highest values for ECF. This could be due to the nature and design of the respective IRP as well as the morphology of IDR [7, 8]. In contrast to the SPA, the new SPA+ variant has more and much longer elastic fingers (Fig. 1). These could lead to increased effectiveness due to their better adaptability. At the same time, the increased amount of material because of bigger lamellae in the interdental space during the cleaning process also increases the ECF and reduces the flexibility of gliding through the respective IDR morphology (Fig. 4).

To date, we are not aware of any other studies evaluating the in-vitro testing of different IDR care products according ECF approximating the real conditions of the oral cavity. Using AS results in a much wider range of uses of IRPs for IDR, especially by applying larger sizes, with reduced ECF and could build the bridge between good in-vivo outcomes of IRP for gingivitis reduction [1] and the lower in-vitro results. It could be hypothesized that this will be associated with (1) increased acceptance by the patients, who thus become more motivated to perform regular interdental hygiene at home and (2) higher variability of IRP sizes for better cleaning efficacy which both has to be confirmed by future clinical investigations.

## Limitations

Despite the attempt to adapt our experimental model constantly to the conditions in the oral cavity, the study remains an in-vitro experiment, which can only be compared with the real conditions in the patient to a limited extent as discussed in extension previously by our group [7]. The exclusively straight brushing movements, which we have retained for the purpose of comparability of the results, do not occur in this way in the context of home oral hygiene. Furthermore, it was no longer possible for us to measure the effectiveness of biofilm reduction when using artificial saliva. Nevertheless, the composition of the saliva differs between patients and can thus lead to altered results. Thus, to prove the current in-vitro results, clinical investigation has to evaluate the cleaning efficacy.

## Conclusion

Within the limitation of the current in-vitro study, the extension of our model to include testing with AS allowed a more realistic assessment of IRP and showed significantly reduced ECF for IRPs used with AS. It is left to further clinical studies to find out whether the effectiveness, i.e., the reduction of microbial plaque in the IDR, is also influenced.

## Data Availability

The datasets used and/or analyzed during the current study are available from the corresponding author on reasonable request.

## Abbreviations

AS: artificial saliva
IDB: interdental brushes
IRP: interdental rubber picks
IDR: interdental area
ECF: experimental cleaning force
